# Editorial: Community series - Purple Haze: issues on cannabis legalization, volume II

**DOI:** 10.3389/fpsyt.2023.1241229

**Published:** 2023-07-21

**Authors:** Stéphane Potvin, Yasser Khazaal, Amine Benyamina, Marc N. Potenza

**Affiliations:** ^1^Centre de recherche de l'Institut Universitaire en Santé Mentale de Montréal, Montreal, QC, Canada; ^2^Department of Psychiatry and Addiction, University of Montreal, Montreal, QC, Canada; ^3^Addiction Medicine, Department of Psychiatry, Lausanne University Hospital and Lausanne University, Lausanne, Switzerland; ^4^Department of Psychiatry and Addictology, Hospital Paul Brousse Villejuif, Unité Psychiatrie-Comorbidités-Addictions-Unité de Recherche PSYCOMADD 4872, Université Paris Saclay, Villejuif, France; ^5^Connecticut Mental Health Center, New Haven, CT, United States; ^6^Connecticut Council on Problem Gambling, Wethersfield, CT, United States; ^7^Wu Tsai Institute, Yale University, New Haven, CT, United States; ^8^Department of Psychiatry, Child Study and Neuroscience, Yale School of Medicine, New Haven, CT, United States

**Keywords:** cannabis, legalization, harm reduction, public health, policy

Considering the progressive legalization of cannabis across jurisdictions, we prepared a Research Topic that addresses significant issues relevant for future legalization initiatives. This Research Topic follows a first Research Topic on the same theme (https://www.frontiersin.org/research-topics/11986/purple-haze-issues-on-cannabis-legalization). The current Research Topic seeks to: (i) document the psychiatric and cognitive consequences of cannabis products, used either for recreational or medical purposes; (ii) document the impacts of cannabis legalization in North America, with special attention to youth, emergency department visits and sex/gender differences; (iii) provide a framework for medical cannabis administration; and (iv) define priority areas deserving more research.

Among the potential harms of cannabis, the association with psychosis is one of the issues that has received the most attention in the context of recreational cannabis legalization. As Hall points out, the nature of this association is too often judged by the authors' preconceptions about the merits of recreational marijuana legalization (RML), with opponents of RML being prone to assert the causal nature of the association, and supporters being inclined to deny it. In his article, Hall shows that the literature is sufficiently robust to support a causal interpretation of the association between cannabis and psychosis. With nuance, he argues that one can nonetheless be in favor of RML, as taking a stance on such a policy requires considering all its advantages and disadvantages, and that presumed advantages (example: reducing the share of the illegal market, increasing potential access to prevention) may outweigh its disadvantages.

In addition to psychosis, the impact of cannabis on youth is another major concern. In their scoping review, Kaur et al. analyzed 140 studies to determine whether cannabis produces more harm in young people. The available literature shows that initiating cannabis use at a younger age is clearly associated with worse outcomes for psychosis and cannabis use disorder. Regarding depression and suicidality, the evidence is mixed, and there is a relative lack of data in the case of anxiety. Despite the methodological limitations of the studies (e.g., uncontrolled confounding factors), the authors argue that there is sufficient evidence to recommend to delay as much as possible the age of initiation of cannabis use.

The actual impact of legalizing cannabis for recreational use remains a controversial topic. To shed some light on the subject, Athanassiou et al. performed a systematic review of studies published to date. As quality criteria, the authors selected only longitudinal studies that compared key public health outcomes between regions (e.g., States) that had or had not legalized the substance. Thirty-two studies were identified showing that RML in the United States is associated with increases in the prevalence of cannabis use in adults, increases in healthcare-related service use, increases in traffic fatalities, increases in alcohol use, no change in cigarette use and an unexplained decrease in opioid prescriptions. The potential impact on RML on crime and suicide were insufficiently studied.

In the Canadian context, Rubin-Kahana et al. examined the impact of RML on youth. To date, the data do not suggest a marked increase in consumption among young people. On the other hand, preliminary results suggest a potential increase in hospitalizations and emergency department (ED) visits among young people, but these trends remain to be confirmed. In the future, research will need to pay close attention to high-potency cannabis use among young people. In a complementary manuscript, Matheson and Le Foll discussed the effects of RML as a function of sex and gender. Cannabis has traditionally been more prevalent in men than women. However, the gap between men and women is narrowing. It is not known whether RML may have played a role in this trend. It is important to bear in mind, however, that in general, men have a more favorable opinion of RML, and perceive less harm associated with the substance. Matheson and Le Foll highlight the lack of data on sex and gender differences in car accidents and hospitalizations, and the need to study the impact of RML on trans-gender and gender-diverse populations.

In an article devoted to cannabis-related ED visits, Crocker et al. observed an increase in ED visits following the legalization of recreational cannabis use. Although these events are not frequent, cannabis-related ED presentations are complex; hence, it would be relevant to make brief interventions available to those affected. Furthermore, with the increase in delta-9-tetrahydrocannabinol (Δ^9^-THC) content in cannabis, the authors mention that it is reasonable to anticipate that the prevalence of cannabis-related ED visits could increase in the future.

The understanding of the impacts of RML extend beyond legal changes. The unfolding of changes in policy also critically matter. In Canada, the province of Ontario had the highest number of in-person retail stores 3 years after RML in October 2018. Using data from the *Ontario Cannabis Store*, Tassone et al. draw a detailed portrait of the products sold in this province. Beyond the diversity of products, what is particularly striking is that most inhaled products have concentrations of Δ9-THC higher than 20%. There is growing evidence that high-potency cannabis produces more harm (1). However, this literature is based on a cut-off of 10% to classify cannabis as having high-potency. These data illustrate the urgent need to update our knowledge on cannabis with a potency higher than 20%.

Regarding the legalization of cannabis for medical purposes, there are no clinical guidelines to follow. In order to fill this gap, Maccallum et al. propose a practical framework providing general recommendations relating to modes of administration, compounds contained in cannabis, dosage, frequency of administration, characteristics of treated individuals and drug-drug interactions. In an article on the hepatic metabolism of ingested cannabinoids, Smith and Gruber review drug-drug interactions. They discuss interactions between cannabidiol and certain drugs including anti-epileptics and antidepressants. Among the areas to be investigated in the future, the authors highlight the need to better understand interactions involving minor cannabinoids such as cannabinol.

Finally, in an article on the addictive potential of medical cannabis, Cooke et al. documented the number of people who developed a cannabis use disorder (CUD) during a clinical trial lasting 12 months (*n* = 163). Worryingly, they observed that 11.7% of participants and 17.1% of people with (nearly-) daily use developed a CUD during the intervention. The addictive potential of medical cannabis will require close monitoring in the future.

With increasing legalization in different jurisdictions, it is vital to allocate resources for research, prevention, treatment, and policy initiatives. In light of the findings discussed in this topic, it is important to establish ongoing monitoring and seize this opportunity to develop new prevention and harm reduction strategies.

## Author contributions

SP wrote the manuscript. YK, AB, and MP provided critical comments. All authors approved the final version of the manuscript.
